# The Potential Beneficial Effect of EPA and DHA Supplementation Managing Cytokine Storm in Coronavirus Disease

**DOI:** 10.3389/fphys.2020.00752

**Published:** 2020-06-19

**Authors:** Zoltán Szabó, Tamás Marosvölgyi, Éva Szabó, Péter Bai, Mária Figler, Zsófia Verzár

**Affiliations:** ^1^Faculty of Health Sciences, Institute of Nutritional Sciences and Dietetics, University of Pecs, Pecs, Hungary; ^2^Medical School, Institute of Bioanalysis, University of Pecs, Pecs, Hungary; ^3^Department of Biochemistry and Medical Chemistry, Medical School, University of Pecs, Pecs, Hungary; ^4^Department Medical Chemistry, Faculty of Medicine, University of Debrecen, Debrecen, Hungary; ^5^MTA-DE Lendület Laboratory of Cellular Metabolism, Debrecen, Hungary; ^6^Faculty of Medicine, Research Center for Molecular Medicine, University of Debrecen, Debrecen, Hungary; ^7^2nd Department of Internal Medicine and Nephrology Centre, Clinical Centre, University of Pecs, Pecs, Hungary

**Keywords:** COVID-19, DHA – 22:6n-3, EPA - 20:5n-3, supplementation, IL-6 (Interleukin 6), IL-1ß

In the recent COVID-19 (caused by SARS-Cov-2 virus) pandemic a subgroup of patient death is attributed to the so-called “cytokine storm” phenomenon (also called cytokine release syndrome or macrophage overactivation syndrome) (Mehta et al., 2020). To date, the molecular events that precipitate a “cytokine storm” or the applicable therapeutic strategies to prevent and manage this process is not elucidated because of the complex nature of this problem (Tisoncik et al., 2012). Recent articles suggest that specific nutrients such as vitamin B_6_, B_12_, C, D, E, and folate; trace elements, including zinc, iron, selenium, magnesium, and copper may play a key role in the management of cytokine storm (Calder et al., 2020; Grant et al., 2020; Muscogiuri et al., 2020). Among these micronutrients LC-PUFAs (long chain polyunsaturated fatty acids) such as EPA (eicosapentaenoic acid) and DHA (docosahexaenoic acid) are noteworthy because of their direct influence in the immunological response to viral infections (Calder et al., 2020; Messina et al., 2020).

In this paper, we would like to draw the attention to the possible beneficial effect of EPA and DHA supplementation in SARS-CoV-2 infection and urge the medical community for further investigations and conduction of clinical trials.

Evidence suggests that n-3 LC-PUFAs can modulate the immune response and function in many ways (Calder, 2007, 2013; Zivkovic et al., 2011; Maskrey et al., 2013; Tao, 2015; Allam-Ndoul et al., 2017). Among these complex immunomodulatory effects, interleukin-6 (IL-6) and interleukin-1ß (IL-1β)—because of the suspected central regulatory role in the “cytokine storm”—should be highlighted. These cytokines can be affected by dietary EPA and DHA intake (Figure 1). In addition, poly(ADP-ribose) polymerase enzymes that have anti-inflammatory properties, translatable to human COVID-19 infection were shown to improve tissue levels of DHA and EPA, as well as the downstream anti-inflammatory metabolites of EPA and DHA (Kiss et al., 2015; Curtin et al., 2020) further underscoring the applicability of DHA and EPA in COVID-19.

**Figure 1:**
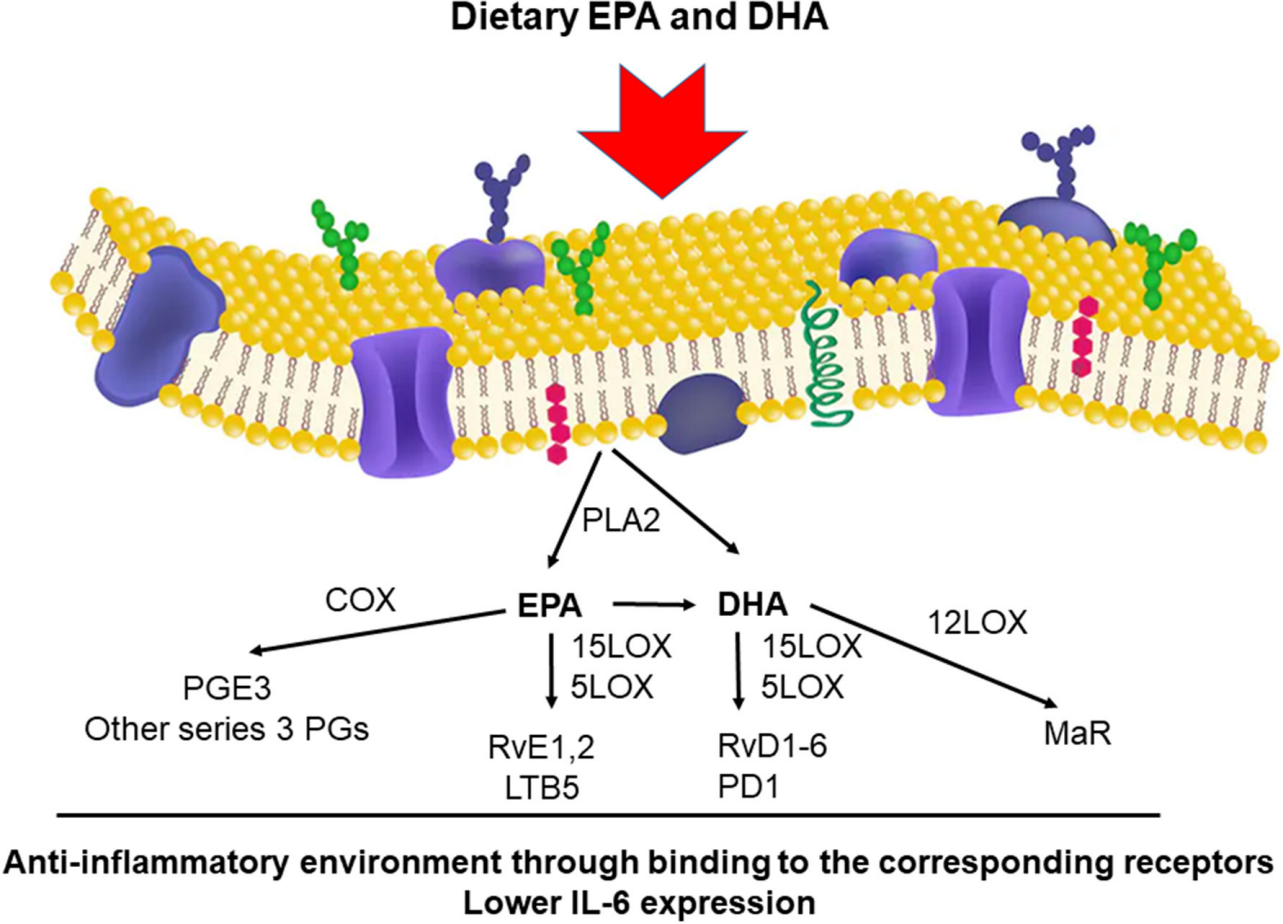
Main pathways for the metabolism of DHA and EPA yielding anti-inflammatory metabolites. The two most important n-3 LCPUFAs, DHA and EPA, can be either released from the membrane of cells by PLA2 or dietary DHA and EPA can be utilized for enzymatic conversion by LOX and COX enzymes that generate bioactive, anti-inflammatory downstream metabolites. These metabolites bind to their respective receptors and elicit anti-inflammatory changes in cells mainly through rearranging the transcriptome. These pleiotropic effects altogether lead to decreases in IL-6, IL-1, or TNFα, key cytokines provoking cytokine storm. PLA2, phospholipase A2; EPA, eicosapentaenoic acid; DHA, docosahexaenoic acid; LOX, Lipoxygenase; PGs, Prostaglandins; PGE3, Prostaglandin E3; RvE1,2, Resolvin E1 and E2; LTB5, Leukotriene B5; RvD1-6, D-series resolvins; PD1, Protectin D1; MaR, Maresin. The sketch of the membrane is a stock image from shutterstock.com (No. 1106910629).

IL-6 blockade using Tocilizumab monoclonal antibody has been identified as a feasible therapeutic target in SARS-CoV-infections (Liu et al., 2020), nevertheless, reducing the expression of additional proinflammatory cytokines (e.g., IL-1ß, IL-38) may have beneficial effects (Conti et al., 2020).

Both EPA and DHA can decrease the secretion of inflammatory cytokines *in vitro* and animal studies (Gutierrez et al., 2019). Pre-supplementation with DHA (400 mM) significantly decreased the release of IL-6 and IP-10 by Calu-3 cells infected with Rhinovirus RV-43 and RV-1B (Saedisomeolia et al., 2009).

Based on the results of a randomized, controlled study published in 2018, high-dose (1.5 g/day EPA and 1.0 g/day DHA) n-3 supplementation can reduce plasma levels of both IL-6 and IL-1ß (Tan et al., 2018). The anti-inflammatory effect of EPA and DHA supplementation seems consistent with most of the previous clinical findings (Fritsche, 2006; Vedin et al., 2008; Kiecolt-Glaser et al., 2012; Muldoon et al., 2016; Calder et al., 2020) (Table 1).

**Table 1:** The effects of DHA and EPA supplementation on cytokine production.

**References**	**Type**	**Supplementation**	**Subjects**	**Effects**
Ramon et al. (2012)	*in vitro*	^a^50 nM 17-hDHA ^b^100 nM 17-hDHA	CD19^+^ B cells	IL-6 ↓44%^a^ IL-10 ↓49 %^a;^ 54%^b^ TNF-α
Allam-Ndoul et al. (2017)	*in vitro*	^a^10 μM DHA ^b^50 μM DHA ^c^75 μM DHA ^d^10 μM EPA ^e^50 μM EPA ^f^75 μM EPA	THP-1 acute monocytic leukemia cell line	IL-6 ↓ 12%^a^; 19%^b^; 30%^c^; 6%^d^; 13%^e^; 24%^f^ TNF ↓ 6%^a^; 12%^b^; 15%^c^; 18%^f^
Saedisomeolia et al. (2009)	*in vitro*	^a^200 μM DHA ^b^400 μM DHA ^c^200 μM EPA ^d^400 μM EPA	Airway epithelial cells (Calu-3) with RV-43	IL-6 ↓ 16%^b^ IL-8 IP-10 ↓ 28%^b^
			Airway epithelial cells (Calu-3) with RV-1B	IL-6 ↓ 13%^a^; 29%^b^ IL-8 IP-10 ↓ 24%^b^
Tan et al. (2018)	RCT	^a^1.5 g/day DHA 4th weeks ^b^1.5 g/day DHA 8th weeks	Plasma of patients with chronic venous leg ulcers	IL-6 ↓ 12%^a^; 22%^b^ IL-1ß ↓ 29%^a^; 44%^b^ TNF-α ↓ 12%^a^; 23%^b^
Vedin et al. (2008)	RCT	1.7 g/day DHA and 0.6 g/day EPA	Blood mononuclear leukocytes of Alzheimer disease patients	IL-6 ↓ 43% IL-1ß ↓ 35% TNF-α
Kiecolt-Glaser et al. (2012)	RCT	^a^2.5 g/day n-3 PUFAs ^b^1.25 g/day n-3 PUFAs	Serum of healthy adults	IL-6 ↓^a, b^ TNF-α ↓^a, b^
Zhou et al. (2019)	RCT	^a^3.6 g/day EPA + DHA ^b^1.8 g/day EPA + DHA	Peripheral blood mononuclear cells (PBMCs) in Hypercholesterol-emic Adults	TG ↓ 20%^a;^ 13 %^b^ IL-6 ↓ 37%^a^; TNF -α
Muldoon et al. (2016)	RCT	0.4 g/day DHA and 1.0 g/day EPA	Serum of healthy adults	IL-6

*% change in the expression of cytokines upon DHA and/or EPA supplementation were either calculated from original data or reproduced from given publications, where available. A ↓ notation stands for a statistically significant decrease in the measured levels of the examined cytokines. Identical superscripts both in the “Supplementation” and “Effects” columns (a, b, c, d, e, f) denote the published effect(s) of the given supplementation group/dose*.

A DHA metabolite (17-hDHA) can reduce IL-6 secretion in human B cells (Ramon et al., 2012).

The triglyceride-lowering effect of n-3 LC-PUFA supplementation is well-known (Yanai et al., 2018; Zhou et al., 2019; Abdelhamid et al., 2020). Lower levels of triglyceride present a lower risk of developing a “cytokine storm” based on the score from the available sHLH score system (Mehta et al., 2020). This approach represents another standpoint for the promotion of n-3 LC-PUFA supplementation in COVID-19 disease.

In addition, evidence suggests that in non-viral infected critically ill patients n-3 LC-PUFA supplementation can be helpful but data are highly limited (Rangel-Huerta et al., 2012). A recent meta-analysis reported the effects of omega-3 fatty acids and/or antioxidants in adults with acute respiratory distress syndrome in which the authors concluded that any beneficial effect in the duration of ventilator days and ICU length of stay or oxygenation at day 4 seems uncertain because of the very low quality of evidence (Dushianthan et al., 2019). To date there is no direct evidence of any beneficial or deleterious effect of immunonutrition with EPA and DHA in COVID-19 patients.

EPA and DHA supplementation can alter many biological pathways which may have direct influence in the outcome of COVID-19 (Fenton et al., 2013; Duvall and Levy, 2016; Curtin et al., 2020).

The safety of EPA and DHA supplementation should be also highlighted. Although, the US Department of

Health & Human Services National Institutes of Health Office of Dietary Supplements (ODS) concluded that a daily intake of EPA+DHA of up to 3.0 g/d is safe (Usdhhs N. I. O. H. and Office of Dietary Supplements, 2019), the European Food Safety Authority (EFSA) stated that the long-term consumption of EPA and DHA supplements at combined doses of up to about 5 g/day appears to be safe for the general public (EFSA, 2012). In addition some evidence suggest that long-term supplementation of EPA and DHA may have side effects such as increasing risk of certain types of cancers, but the results are conflicting (Gerber, 2012; Alexander, 2013; Serini and Calviello, 2018). It should be also noticed that the usage of algae- or plant-based sources of EPA and DHA seems more preferable than marine or animal-based sources (Doughman et al., 2007; Lane et al., 2014; Harwood, 2019).

Summary: Based on the available data, the supplementation of EPA and DHA in COVID-19 patients appears to have potential beneficial effect in managing the “cytokine storm.” Therefore, the use of EPA and DHA supplementation should be considered as both a supportive therapy and a prevention strategy in SARS-Cov-2 infection.

## Author Contributions

ZS, TM, and ÉS drafted the manuscript. TM, PB, and ZS designed the figure and the table. MF, ZV, and ÉS substantial contributions to the conception by supervising all the processes. PB, MF, ZV, and ÉS revised the manuscript critically for important intellectual content. TM and ZS drafted the reference list. ZS and TM proofread the final manuscript. All authors agree that our work is accountable for all aspects of the work in ensuring that questions related to the accuracy or integrity of any part of the work are appropriately investigated and resolved. All authors read and approved the final manuscript.

## Conflict of Interest

The authors declare that the research was conducted in the absence of any commercial or financial relationships that could be construed as a potential conflict of interest.
